# BRG1 establishes the neuroectodermal chromatin landscape to restrict dorsal cell fates

**DOI:** 10.1126/sciadv.adj5107

**Published:** 2024-03-01

**Authors:** Jackson A. Hoffman, Ginger W. Muse, Lee F. Langer, A. Isabella Patterson, Isabella Gandara, James M. Ward, Trevor K. Archer

**Affiliations:** ^1^ Epigenetics and Stem Cell Biology Laboratory, National Institutes of Environmental Health Sciences, Research Triangle Park, NC 27709, USA.; ^2^ University of North Carolina at Chapel Hill, Chapel Hill, NC 27599, USA.; ^3^ Integrative Bioinformatics, National Institutes of Environmental Health Sciences, Research Triangle Park, NC 27709, USA.

## Abstract

Cell fate decisions are achieved with gene expression changes driven by lineage-specific transcription factors (TFs). These TFs depend on chromatin remodelers including the Brahma-related gene 1 (BRG1)-associated factor (BAF) complex to activate target genes. BAF complex subunits are essential for development and frequently mutated in cancer. Thus, interrogating how BAF complexes contribute to cell fate decisions is critical for human health. We examined the requirement for the catalytic BAF subunit BRG1 in neural progenitor cell (NPC) specification from human embryonic stem cells. During the earliest stages of differentiation, BRG1 was required to establish chromatin accessibility at neuroectoderm-specific enhancers. Depletion of BRG1 dorsalized NPCs and promoted precocious neural crest specification and enhanced neuronal differentiation. These findings demonstrate that BRG1 mediates NPC specification by ensuring proper expression of lineage-specific TFs and appropriate activation of their transcriptional programs.

## Teaser

BRG1 depletion alters neural progenitor cell fates by disrupting chromatin dynamics during differentiation.

## INTRODUCTION

Access to DNA is regulated by large multisubunit chromatin remodeling complexes that work in concert with many nuclear processes to ultimately regulate gene expression and cell identity. The Brahma-related gene 1 (BRG1)-associated factor (BAF; also known as SWI/SNF) complex uses adenosine 5′-triphosphate hydrolysis by the adenosine triphosphatase BRG1 to slide or eject histone octamers to remodel nucleosomes (*1*). In general, the BAF complex is required to maintain chromatin accessibility at promoters and regulatory elements to facilitate transcription factor and transcriptional machinery interactions (*2*, *3*). Hence, the BAF complex is associated with and often required for active transcription.

Understanding how BAF complexes regulate cell identity and cell fate transitions is of great interest to human health research. The BAF complex is also essential throughout development, and ablation of *SMARCA4* (or many other BAF subunit genes) results in embryonic lethality (*4*). It is estimated that >20% of human cancers contain mutations in at least one BAF subunit (*5*, *6*), and protein expression of the catalytic subunit BRG1 (encoded by the *SMARCA4* gene) is lost in >40% of brain, liver, kidney, and intestinal cancers (*7*). Conversely, up-regulation of BAF subunits can also have oncogenic effects, and high levels of BRG1 expression are found in a variety of tumor types and often associated with poorer prognoses (*8*–*10*). Thus, the activity and expression of the BAF complex are essential, and there are context-dependent requirements for BAF subunits throughout development and in tumorigenesis.

Mutations in *SMARCA4* or *SMARCB1* (which encodes the core subunit BAF47) can give rise to highly aggressive pediatric brain tumors such as atypical teratoid/rhabdoid tumors (ATRTs) and medulloblastomas (*11*–*17*). In the case of ATRTs, the requirement for BAF47 is strongly established, and *SMARCB1* mutations are found in the vast majority of tumors (*18*). *SMARCA4* mutations in ATRT are rarer and associated with earlier onset and worse prognoses (*19*–*21*). As these tumors are thought to arise from progenitor cells during nervous system development, a comprehensive understanding of BAF complex functions during neuroectodermal differentiation is critical, and human embryonic stem cells (hESCs) provide an apt model system for investigation.

Individual BAF subunits have a variety of context-dependent requirements during hESC self-renewal and differentiation in both human and mouse (*22*). Previously, we found that BAF47 is essential for neuroectodermal differentiation of human hESCs, during which it is required to regulate enhancer accessibility (*23*). Knockdown (KD) of either BRG1 or BAF47 in hESCs leads to derepression of bivalent lineage-specific genes, suggesting that the BAF complex safeguards the pluripotent state of self-renewing hESCs (*23*, *24*). During neuroectodermal differentiation, the BAF complex undergoes a series of tightly regulated paralog switching events, with distinct subunit compositions defining hESC-, neural progenitor–, and neuron-specific complexes (*25*). Mutation of the complex-specific subunits results in corresponding defects in pluripotency, differentiation, and neural morphogenesis and function (*26*–*30*). In general, conditional deletion of BAF subunits during specific stages of neural differentiation leads to pleiotropic effects on proliferation and specification of neural cell types (*30*, *31*). Throughout these processes, the BAF complex can interact with a variety of lineage-determining neural transcription factors such as SOX2, PAX6, and ASCL1 (*32*–*35*).

We set out to directly assess the requirement for the BRG1 subunit and BAF complex activity in self-renewing hESCs and during neural progenitor cell (NPC) specification. By depleting BRG1 with either short hairpin RNA (shRNA) expression or proteolysis targeting chimera (PROTAC) treatment, we demonstrate that BAF complex activity is essential for proper specification of NPCs at the onset of neuroectodermal differentiation. BRG1 depletion resulted in the dorsalization of NPCs, enhanced and altered neuronal differentiation, and precocious neural crest differentiation. BRG1 was required to establish and maintain accessibility at enhancers that were activated during the initial stages of differentiation. These BRG1-dependent enhancers were highly enriched for neuroectodermal lineage transcription factor motifs. Thus, we concluded that BAF complex activity is essential for the establishment of the NPC chromatin landscape by lineage-specific transcription factors.

## RESULTS

### BRG1 controls neurectoderm developmental transcription programs in hESCs

Inducible shRNA depletion of BAF47 protein in H1 hESCs resulted in widespread derepression of gene expression and inhibition of directed neuroectodermal differentiation (*23*). This demonstrated a critical role for the BAF47-containing BAF complexes in hESCs and during differentiation. However, BRG1 immunoprecipitation followed by mass spectrometry revealed that BAF complex assembly was not disrupted by the reduction of BAF47 protein (fig. S1). Independent of BAF47 depletion, BRG1 specifically pulled down BAF subunits from all three variants of the BAF complex (fig. S1). Thus, it was unclear whether the changes observed in BAF47KD hESCs were the result of altered, reduced, or mistargeted BAF complex activity.

**Fig. 1.**
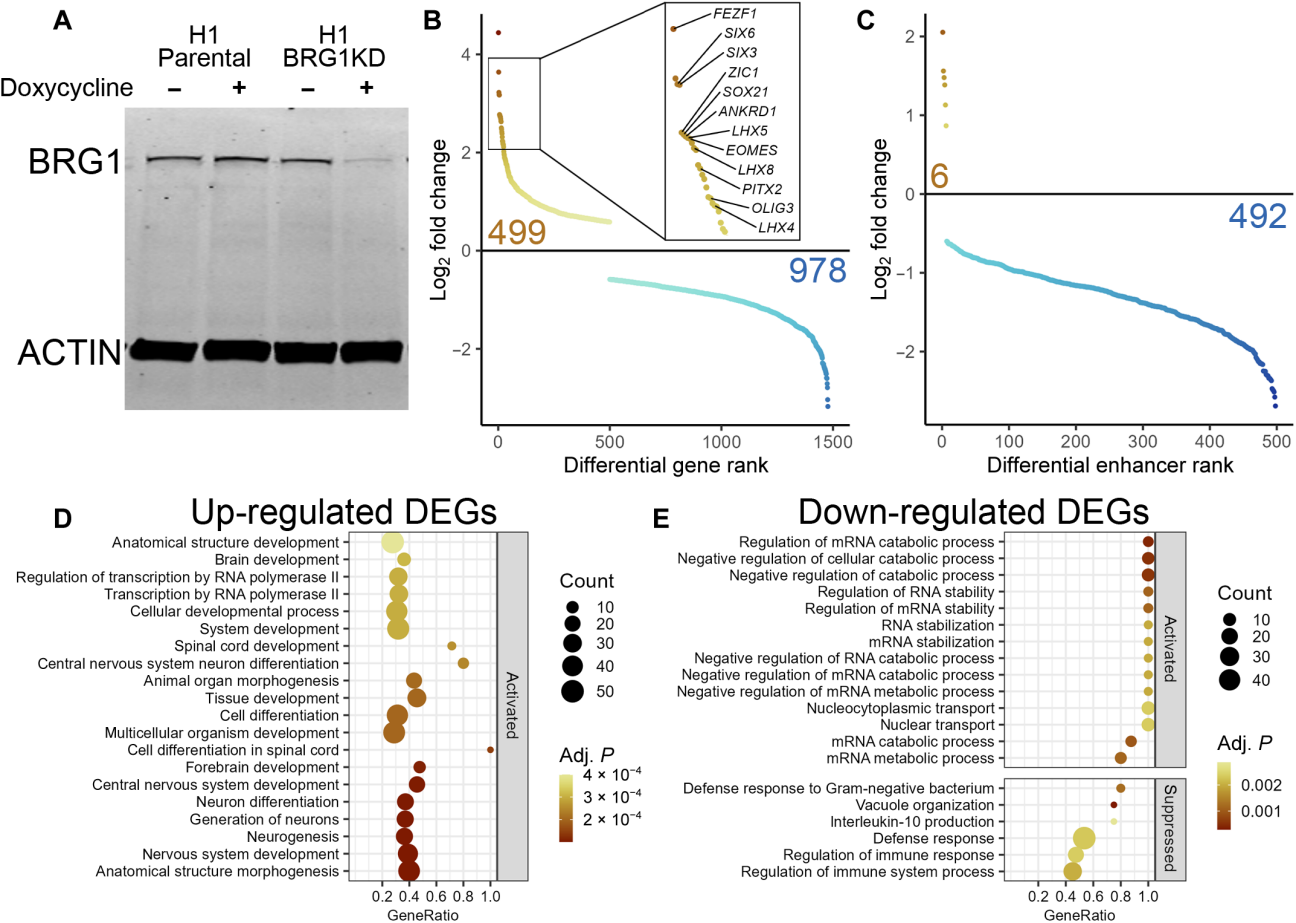
BRG1 controls neurectoderm developmental transcription programs in hESCs. (**A**) Western blot for BRG1 and actin protein levels in parental and BRG1KD cells ± Dox. (**B**) Mean RNA sequencing (RNA-seq) log_2_ fold change of BRG1KD DEGs ranked in descending order. Inset highlights select up-regulated neuroectodermal transcription factors. See also fig. S2. (**C**) Mean RNA-seq log_2_ fold change of BRG1KD differentially expressed enhancers ranked in descending order. (**D**) Gene set enrichment plot of basic processes enriched in up-regulated BRG1KD DEGs. (**E**) Gene set enrichment plot of basic processes enriched in down-regulated BRG1KD DEGs. In (D) and (E), GeneRatio represents the fraction of genes in each biological pathway that are present in the DEG list. “Activated” and “suppressed” panels designate whether the changes in expression of the DEGs are consistent with activation or suppression of the enriched biological pathway.

To directly investigate the requirement for BAF complex activity in hESCs and neuroectodermal differentiation, we generated an H1-derived cell line with inducible expression of *SMARCA4* shRNA (BRG1KD cells). Doxycycline (Dox) induction of *SMARCA4* shRNA expression for 72 hours resulted in 80 to 90% depletion of BRG1 protein levels minimal impact on the expression of pluripotency factors without the deleterious effects reported in studies with prolonged KD of BRG1 (Fig. 1A and fig. S2) (*24*). BRG1 depletion resulted in 1477 differentially expressed genes (DEGs; fold change > 1.5, adjusted *P* < 0.05) (Fig. 1B and fig. S2). Two-thirds of these DEGs were down-regulated after BRG1 depletion, consistent with the association of BRG1 activity and transcriptional activation. Despite this bias toward a requirement for transcriptional activation, 499 genes were up-regulated following BRG1 depletion. BRG1 depletion also significantly altered the transcriptional activity at 498 hESC enhancers (*36*), with nearly all of these being down-regulated (Fig. 1C). Thus, BRG1 was predominantly required to promote gene expression in hESCs.

Previously, we showed that transcription factors involved in neural development such as *ZIC1*, *SOX21*, and *FEZF1* were among the most highly up-regulated genes in BAF47KD hESCs (*23*). These genes were among 12 transcription factors relevant to neuroectodermal differentiation within the top 25 genes up-regulated in BRG1KD hESCs (Fig. 1B, inset, and fig. S2). In addition, the up-regulated DEGs were significantly enriched for biological pathways corresponding to activation of neural differentiation and development (Fig. 1D). Conversely, down-regulated DEGs were enriched for pathways involved in mRNA metabolism and immune response (Fig. 1E). Thus, consistent with previous studies, the transcriptomic changes we observed in BRG1KD hESCs suggested that BRG1 and the BAF complex may also play a role in neuroectodermal differentiation.

### BRG1 depletion promotes formation of atypical NPC populations

**Fig. 2.**
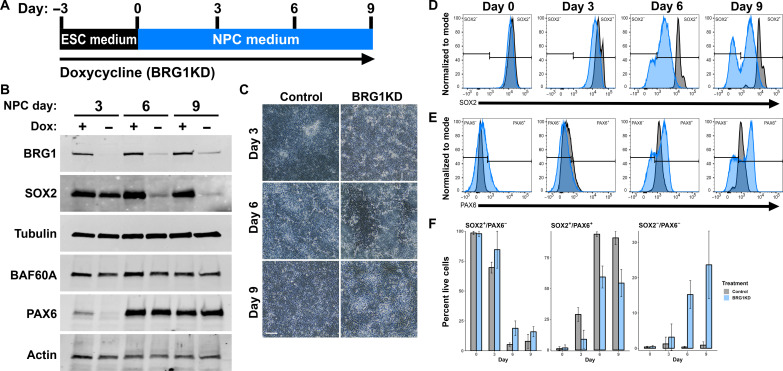
BRG1 depletion promotes formation of atypical NPC populations. (**A**) Graphical depiction of experimental setup for (B) to (F). (**B**) Western blots showing BRG1, SOX2, tubulin, BAF60A, PAX6, and actin protein expression at collection time points. See also figs. S2 and S3. (**C**) Phase contrast images of control and BRG1KD NPCs. Scale bar, 100 μm. (**D** and **E**) Fluorescence-activated cell sorting (FACS) histogram of SOX2 (D) and PAX6 (E) protein detection at collection time points. Log_10_ fluorescent signal depicted for control cells in gray and BRG1KD cells in light blue. Data represents the total of all biological replicates (*n* ≥ 3). (**F**) Graphs depicting the percent of cells at each collection time point that were SOX2^+^/PAX6^−^, SOX2^+^/PAX6^+^, or SOX2^−^/PAX6^−^. Bar height indicates mean percentages, and error bars represent SD of biological replicates (*n* ≥ 3). Control values are in gray, and BRG1KD values are in light blue.

To interrogate the effect of BRG1 depletion on neuroectoderm differentiation, we used dual SMAD inhibition (*37*) to induce the formation of NPCs over the course of a 9-day differentiation protocol (Fig. 2A). To ensure depletion of BRG1 protein levels at the onset of differentiation, BRG1KD hESCs were pretreated with Dox for 72 hours and then split into neural induction medium (NPC medium). Dox was maintained in NPC medium throughout the protocol, ensuring ongoing depletion of BRG1 protein (Fig. 2B). Both control and BRG1KD cells exhibited near complete loss of *POU5F1* and *NANOG* expression by day 3 in NPC medium, indicating that BRG1KD cells were able to successfully exit pluripotency and begin differentiating (fig. S3, B and E). In contrast, we previously showed that despite similar up-regulation of neuroectodermal transcription factors, BAF47KD hESCs were resistant to neural induction and maintained hESC characteristics (*23*). Thus, BRG1KD was distinct from BAF47KD during the first stages of NPC differentiation.

Over the course of the NPC differentiation protocol, control cells formed a dense lawn of cells (Fig. 2C). By day 6 of differentiation, BRG1KD cultures had a more heterogeneous appearance with frequent regions of cells with mesenchymal morphology (Fig. 2C). This abnormal morphology suggested that BRG1 depletion had altered the normal course of differentiation. NPC differentiation can be tracked by measuring the expression of SOX2 and PAX6, two transcription factors that are critical for NPC specification and maintenance. In control cells, SOX2 was highly expressed in hESCs and maintained at high levels through day 9 of NPC differentiation (Fig. 2D). PAX6 was not expressed in control hESCs and became uniformly expressed by day 6 of NPC differentiation (Fig. 2E). By days 6 and 9, nearly all control cells were positive for both SOX2 and PAX6 (Fig. 2F and fig. S3C). BRG1KD cells also formed populations of PAX6^+^ or SOX2^+^ NPCs; however, BRG1KD atypically resulted in the presence of cells lacking the expression of these two markers. BRG1KD hESCs expressed high levels of SOX2 before differentiation, but levels of SOX2 expression were progressively diminished over the course of NPC differentiation (Fig. 2D). By day 9, two distinct populations of cells formed in BRG1KD cells—a SOX2^+^ population that maintained SOX2 expression (albeit at a lower level than control cells) and a SOX2^−^ population with a complete absence of SOX2 expression (Fig. 2D). SOX2 expression was also markedly reduced in BRG1KD NPCs when detected via Western blot or reverse transcription polymerase chain reaction (RT-PCR) (Fig. 2B and fig. S3C). Similarly, PAX6 was not expressed in BRG1KD hESCs and was up-regulated during NPC differentiation (Fig. 2E). However, instead of a uniformly PAX6^+^ cell population, BRG1KD cells exhibited a broader distribution of PAX6 levels, with a subset of cells expressing higher levels of PAX6 than observed in control cells (Fig. 2E). At days 6 and 9, the majority of BRG1KD cells formed a similar SOX2^+^ PAX6^+^ population with lower levels of SOX2 expression and higher levels of PAX6 (Fig. 2F and fig. S3C). However, more than a third of BRG1KD cells instead formed SOX2^+^/PAX6^−^ or SOX2^−^/PAX6^−^ cell populations (Fig. 2F and fig. S3C). Last, a small and highly variable fraction of BRG1KD cells formed a SOX2^−^/PAX6^+^ population (fig. S3D). Thus, BRG1KD hESCs formed atypical populations of cells during directed NPC differentiation.

### BRG1KD NPCs adopt dorsalized cell fates

**Fig. 3.**
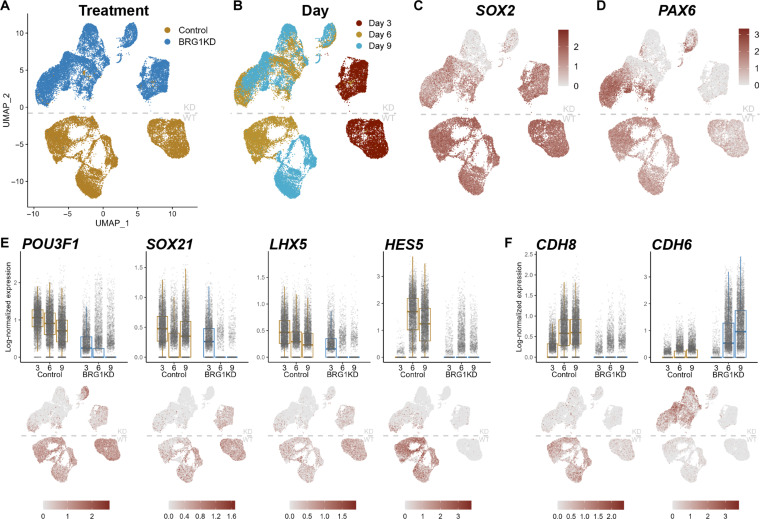
scRNA-seq captures abnormal cell populations in BRG1KD NPCs. (**A** and **B**) UMAP plots depicting clustering of NPCs by treatment (A) and day (B). WT, wild-type. (**C** and **D**) UMAP feature plots depicting *SOX2* (C) and *PAX6* (D) log-scaled expression. (**E** and **F**) Box-and-jitter plots and UMAP feature plots depicting log-scaled expression of neuroectodermal transcription factors (E) and cadherins (F) in control and BRG1KD at days 3, 6, and 9. Each dot represents a single cell, box edges represent the 25th and 75th percentiles, and midline represents the median.

To interrogate the identity of the abnormal NPC populations formed from BRG1KD hESCs, we next performed single-cell RNA sequencing (scRNA-seq) with two biological replicates at days 3, 6, and 9 of NPC differentiation. A high level of consistency was observed between replicates at each time point, so replicates were merged and treated as individual time points for subsequent analyses. Following uniform manifold approximation and projection (UMAP) for dimensional reduction, BRG1KD NPCs formed clusters that were almost entirely distinct from control NPCs (Fig. 3A). Control NPCs from each time point formed defined and separate clusters (Fig. 3B).

Conversely, day 3 BRG1KD NPCs formed a distinct cluster but day 6 and day 9 BRG1KD NPCs formed a single overlapping cluster (Fig. 3B). Similar to our fluorescence-activated cell sorting (FACS) analyses, control NPCs were uniformly positive for both *PAX6* and *SOX2* (Fig. 3, C and D). At day 3, BRG1KD NPCs expressed lower levels of *SOX2* and lacked up-regulation of *PAX6* (Fig. 3, C and D). By days 6 and 9, multiple populations arose in BRG1KD NPCs: (i) *SOX2*^+^/*PAX6*-high cells, (ii) *SOX2*^+^/*PAX6*^−^ cells, and (iii) *SOX2*^−^/*PAX6*^−^ cells (Fig. 3, C and D). Thus, scRNA-seq enabled the identification of the distinct cell populations observed among BRG1KD NPCs.

**Fig. 4.**
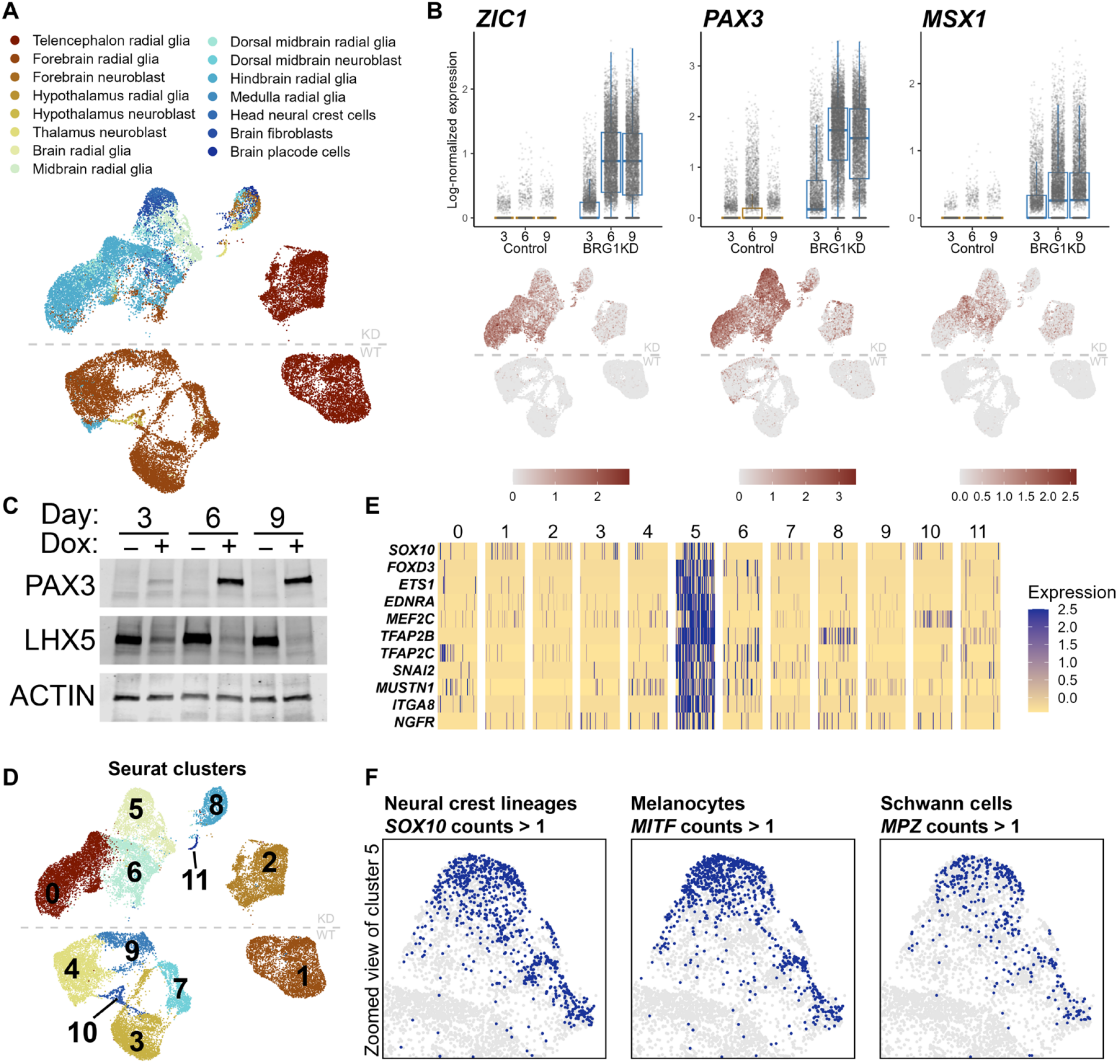
Dorsalization of BRG1KD NPCs and precocious neural crest differentiation. (**A**) UMAP plot depicting first-trimester fetal human brain–based cell type annotations. (**B**) Box-and-jitter plots and UMAP feature plots of log-scaled expression of dorsal neuroectodermal transcription factors at days 3, 6, and 9. Each dot represents a single cell, box edges represent the 25th and 75th percentiles, and midline represents the median. (**C**) Western blots for PAX3 and LHX5 at NPC collection time points. (**D**) UMAP plot showing de novo Seurat cluster assignments. See also figs. S4 and S5. (**E**) Heatmap of log-scaled expression of neural crest cell marker genes in randomly downsampled Seurat clusters (50 cells per cluster). (**F**) Zoomed-in UMAP plots highlighting cells in cluster 5 with more than one count of *SOX10*, *MITF*, and *MPZ*.

Examination of the expression of additional NPC marker genes further revealed the abnormality of BRG1KD NPCs. Most control NPCs expressed transcription factors involved in NPC maintenance and identity such as *POU3F1*, *SOX21*, *LHX5*, and *HES5* (Figs. 3E and 4C). Conversely, expression of these markers was absent from the majority of BRG1KD NPCs (Fig. 3E). Similarly, control NPCs expressed *CDH8*, a cell surface marker of neurogenic progenitors in the developing brain (Fig. 3F) (*38*). Most BRG1KD NPCs lacked *CDH8* expression and instead expressed *CDH6*, a marker of gliogenic progenitors (Fig. 3F) (*38*). Hence, BRG1KD NPCs appeared to be diverging from the typical NPC phenotype toward an alternative cell fate.

To gain further insight into the potential fates of BRG1KD NPCs, we performed cell type annotation using CellTypist (*39*) and an atlas of transcriptional profiles from first-trimester fetal human brains (*40*). Most control NPCs were annotated as telencephalon or forebrain radial glia, demonstrating that control NPCs adopted a ventral or rostral cell fate (Fig. 4A). At day 3, BRG1KD NPCs were also annotated as telencephalon radial glia, indicating that BRG1KD NPCs began differentiation on the same fate trajectory as control NPCs (Fig. 4A). However, at days 6 and 9, BRG1KD NPCs were mostly annotated as midbrain, hindbrain, and medulla radial glia, indicating that BRG1 depletion had resulted in NPCs with a more dorsal or caudal NPC fate. Most day 6 and day 9 BRG1KD NPCs had strongly up-regulated expression of transcription factors associated with the development of the dorsal neural tube such as *ZIC1*, *PAX3*, and *MSX1* (Fig. 4, B and C, and fig. S3E). Unexpectedly, a subset of BRG1KD NPCs were also annotated as “head neural crest cells” and “brain placode cells” (Fig. 4A). This further indicated that BRG1KD NPCs had adopted a fate similar to that of cells at the dorsal side of the developing neural tube from which the neural crest arises or the dorsal ectoderm that gives rise to the placodes.

To facilitate the characterization of both control and BRG1KD cell populations, we used de novo clustering to define 11 cell clusters (Fig. 4D). Among BRG1KD-specific clusters, cluster 5 included the BRG1KD cells annotated as neural crest and represented 22% of BRG1KD cells at days 6 and 9. To confirm that these BRG1KD cells had adopted a neural crest fate, we examined marker genes that were specifically enriched in cluster 5. Markers of neural crest cells such as *FOXD3*, *TFAP2B*, and *NGFR* were strongly enriched in cluster 5 (Fig. 4E). A subset of cluster 5 cells were also enriched for *SOX10*, a marker of further neural crest–derived lineages (Fig. 4E). Notably, some BRG1KD cells in cluster 5 also expressed *MITF* and *MPZ*, marker genes of melanocytes and Schwann cells, respectively (Fig. 4F). Thus, BRG1 depletion promoted atypical differentiation of NPCs to neural crest cells and more specialized neural crest–derived cell types.

Cluster 8 (BRG1KD cells) and cluster 10 (control cells) were readily identifiable as clusters that exhibited neuronal gene expression patterns (fig. S4, A and C). Both clusters were specifically enriched for a variety of neuronal lineage markers such as *NHLH1*, *STMN2*, and *ELAVL3*

(fig. S4C). Cluster 10 accounted for 1.7 and 5.1% of control cells at days 6 and 9, respectively (fig. S4B). BRG1KD NPCs appeared to be more prone toward neuronal differentiation, as cluster 8 was comprised of ~10% of BRG1KD cells at days 6 and 9 (fig. S4B). However, the BRG1KD cluster also exhibited differential neuronal gene expression from the control cluster, with reduced/absent expression of genes such as *PRMT8* and *NEUROD4* and increased expression of *ASCL1*, *SNCG*, and *GYG1* (fig. S4, D and E). This demonstrated that BRG1KD NPCs more readily progressed to neuronal fates and formed neuronal cells with atypical gene expression patterns.

Cluster 11 also represented a unique BRG1KD cell cluster with a more advanced cell fate than expected. Composed of 90 cells, this cluster was uniquely enriched for markers of cilia development and morphogenesis such as *CCNO*, *CDC20B*, *RSPH1*, and several genes in the *CFAP* family (fig. S5, A and B). Gene set enrichment analysis confirmed that genes involved in cilium assembly were strongly enriched among cluster 11 marker genes (fig. S5C). Thus, depletion of BRG1 during NPC differentiation permitted ectopic specification of NPC-derived ciliated cells, potentially representative of polarized neuroepithelial cells or ciliated neurons (*41*). Together, cell annotation and cluster identification revealed that BRG1KD NPCs adopted a dorsalized cell fate that uniquely gave rise to neural crest cells and was more prone to neuronal differentiation.

### BRG1 is required during the initial stages of NPC specification

**Fig. 5.**
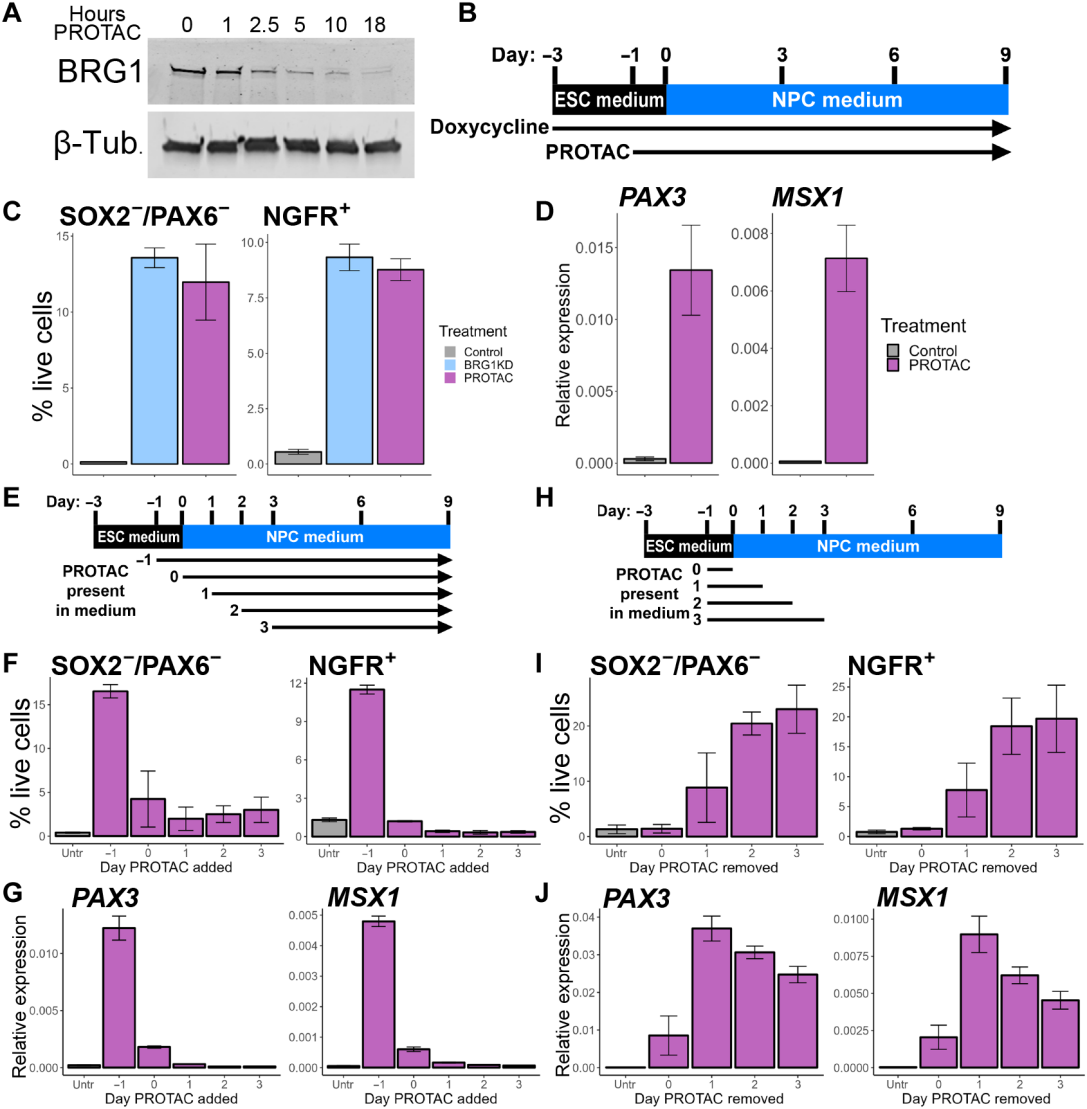
BRG1 is required during the initial stages of NPC specification. (**A**) Western blot of BRG1 and β-tubulin protein levels over a time course of PROTAC treatment. (**B**) Graphical depiction of experimental setup for (C) and (D). See also fig. S6. (**C**) Percent of cells in each treatment that was detected as SOX2^−^/PAX6^−^ and NGFR^+^ by FACS at day 6. (**D**) RT-PCR for *PAX3* and *MSX1* at day 6. (**E**) Graphical depiction of experimental setup for (F) and (G). See also fig. S6. (**F**) Percent of cells in each treatment that was detected as SOX2^−^/PAX6^−^ and NGFR^+^ by FACS at day 9. (**G**) RT-PCR for *PAX3* and *MSX1* at day 9. (**H**) Graphical depiction of experimental setup for (I) and (J). See also fig. S6. (**I**) Percent of cells in each treatment that was detected as SOX2^−^/PAX6^−^ and NGFR^+^ by FACS at day 9. (**J**) RT-PCR for *PAX3* and *MSX1* at day 9. For (C), (F), and (I), bar heights indicate mean percentages, and error bars represent SD of biological replicates (*n* ≥ 3). For (D), (G), and (J), bars depict mean expression level relative to geometric mean of *ACTB*, *GAPDH*, and *TUBA1B*; error bars represent SD of biological triplicates.

We next sought to validate and refine the requirement for BRG1 during early NPC differentiation. We used ACBI1, a BRG1/BRM/BAF180 PROTAC protein degrader, for acute depletion of BRG1 (*42*). In hESCs, BRG1 protein was rapidly degraded by PROTAC treatment (Fig. 5A). We next repeated our NPC differentiation and FACS experiments ±24 hours of PROTAC. PROTAC was maintained in the culture medium for the duration of the differentiation time course (Fig. 5B). PROTAC treatment largely recapitulated the effects of BRG1KD and resulted in nonuniform expression of PAX6 and SOX2 in day 6 NPCs (Fig. 5C and fig. S6, A and B). Similar to BRG1KD NPCs, PROTAC NPCs formed a substantial population of SOX2^−^/PAX6^−^ cells (Fig. 5C and fig. S6B). However, PROTAC NPCs did not form a distinct SOX2^+^/PAX6^−^ population as observed in BRG1KD NPCs (fig. S6B). PROTAC treatment also reproduced the up-regulation of the dorsal transcription factors *PAX3* and *MSX1* observed in BRG1KD NPCs (Fig. 5D). To validate the formation of neural crest cells in BRG1-depleted NPCs, we used the neural crest marker NGFR. In BRG1KD NPCs, 9.3% of cells were NGFR^+^ by flow cytometry, and these NGFR^+^ cells were also SOX2^−^/PAX6^−^ (Fig. 5C and fig. S6, C and E). PROTAC treatment yielded similar results, with NGFR^+^ cells comprising 8.8% of the total number of cells (Fig. 5D and fig. S6D). Together, these experiments demonstrated that the BRG1KD cell populations identified by scRNA-seq could be validated by FACS and largely recapitulated by PROTAC-mediated depletion.

As PROTAC treatment provided for more acute depletion of BRG1 protein, we designed experiments to identify the window during which BRG1 was required for typical dual-SMAD inhibition NPC differentiation. We first designed an experiment to determine whether BRG1 was required at the onset of NPC specification (Fig. 5E). For our standard experimental conditions, PROTAC treatment was initiated 1 day before the switch to differentiation medium. We defined this as addition at day −1 and further defined a series of treatment schemes with the day of PROTAC treatment initiation ranging from day 0 to day 3 (Fig. 5E). Using day 9 as the end point, only the standard “*T* = −1” scheme yielded a robust population of SOX2^−^/PAX6^−^ cells (Fig. 5F and fig. S6, F and G). Beginning PROTAC treatment at later time points resulted in a diminished SOX2^−^/PAX6^−^ population, with at most a third of the cells detected with the −1 time point (Fig. 5F and fig. S6G). Similarly, NGFR^+^ neural crest cells and up-regulation of *PAX3* and *MSX1* were robustly detected when PROTAC was added at day −1, but not detected above control levels in the other treatment schemes (Fig. 5, F and G, and fig. S6, G and H). We thus concluded that the dorsalization of BRG1-depleted NPCs and precocious neural crest specification required BRG1 depletion at the onset of NPC differentiation.

We next examined the duration of PROTAC treatment required to produce the BRG1 depletion phenotype. To do so, we compared control NPCs and NPCs under constant PROTAC treatment to NPCs in which PROTAC was added at day −1 for all samples but then removed at day 0, 1, 2, or 3 (i.e., after 24, 48, 72, or 96 hours of treatment; Fig. 5H). BRG1 protein expression returned to control levels within 24 hours of PROTAC removal (fig. S6G). As with constant treatment, removal of PROTAC at day 2 or day 3 resulted in robust populations of SOX2^−^/PAX6^−^ cells and NGFR^+^ neural crest cells (Fig. 5I and fig. S6, I and J). The size of these cell populations were reduced by more than half when PROTAC was removed at day 1 and completely lost when PROTAC was removed at day 0 (Fig. 5I and fig. S6, I and J). Robust up-regulation of *PAX3* and *MSX1* occurred a day earlier in the time course, with partial induction in the day 0 time point and full induction with the day 1 time point (Fig. 5J). Thus, PROTAC treatment needed to be maintained for at least 48 hours to induce full up-regulation of dorsal markers and for 72 hours to recapitulate the SOX2^−^/PAX6^−^ and NGFR^+^ cell populations and dorsal neural tube transcription factor expression observed with constant treatment. Together, these experiments indicated that BRG1 depletion during the first 48 to 72 hours of NPC differentiation resulted in the formation of a dorsalized NPC population with the capacity to form neural crest cell lineages.

### BRG1 maintains chromatin accessibility and enhancer marks at neuroectoderm enhancers

**Fig. 6.**
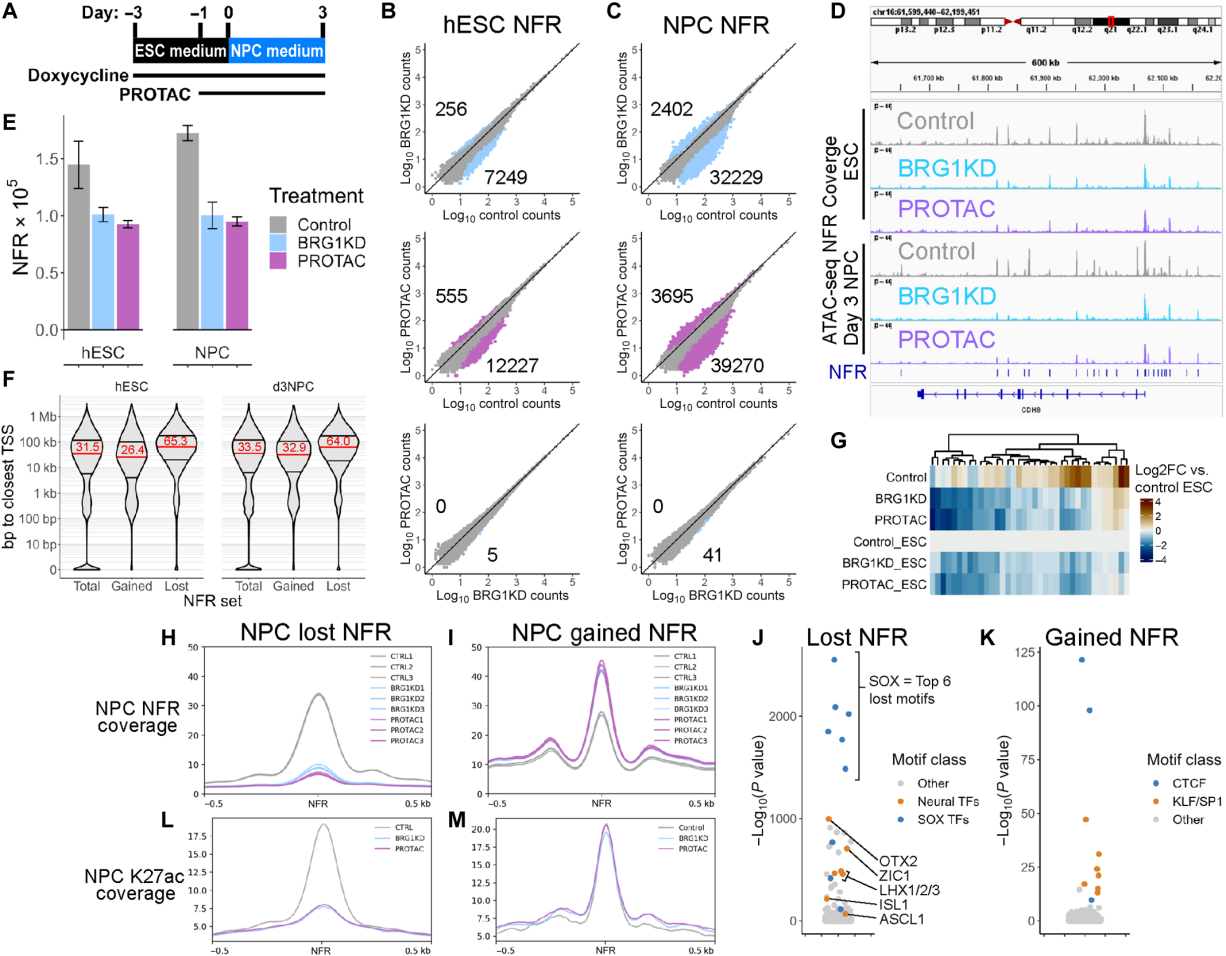
BRG1 maintains chromatin accessibility and enhancer marks at neuroectoderm enhancers. (**A**) Graphical depiction of ATAC-seq experimental setup. Cells were collected at day 0 (ESCs) and day 3 (NPCs). (**B** and **C**) Scatter plots comparing NFR counts between treatments over the total set of NFRs in hESCs (B) and NPCs (C). Colored dots and numbers represent NFRs with significantly more or less accessibility. (**D**) Integrative genomics viewer browser snapshot of ATAC-seq NFR coverage around the *CDH8* gene. NFR peaks are annotated as blue lines above the gene model. Scale for all tracks is 0 to 44. (**E**) Number of NFRs called in each treatment/time point. Bars represent mean, and error bars represent SD of biological triplicates. (**F**) Violin plots of NFR distance from the closest annotated TSS for the total NFR set, NFRs that gain accessibility upon BRG1 depletion (gained) and NFRs that lose accessibility upon BRG1 depletion (lost). Red numbers represent the median distance for each NFR set. (**G**) Heatmap of log_2_ fold change (log_2_FC) relative to control hESCs of NFR coverage over the peaks depicted in (D). (**H** and **I**) Meta-profiles of NFR coverage over lost NFRs (H) and gained NFRs (I). (**J** and **K**) Dot plots of transcription factor motifs enriched in lost NFRs (J) and gained NFRs (K). (**L** and **M**) Meta-profiles of H3K27ac CUT&Tag coverage over lost NFRs (L) and gained NFRs (M).

The BAF complex has typically been associated with open and active regions of chromatin. We thus hypothesized that BRG1 was required to either maintain or establish accessible chromatin at enhancers and/or promoters during the initial stages of NPC differentiation. To test this, we pretreated cells with either 72 hours of Dox to induce BRG1KD or 24 hours of PROTAC to degrade BRG1 protein and maintained these treatments through the first 3 days of NPC differentiation (Fig. 6A). We collected biological triplicates at day 0 and day 3 and performed assay for transposase-accessible chromatin with high-throughput sequencing (ATAC-seq) to identify accessible chromatin regions in hESCs and day 3 NPCs. We limited the ATAC-seq experiment to days 0 and 3 because (i) the PROTAC time course demonstrated that BRG1 was required during the first 2 to 3 days of NPC specification and (ii) both control and BRG1-depleted day 3 NPCs formed single clusters in our scRNA-seq data, suggesting that the cell populations remained uniform at day 3. For analysis of accessible chromatin, nucleosome-free regions (NFRs) were defined as fragments of <100 base pairs, and NFR reads were used to call peaks from each replicate. BRG1KD and PROTAC samples consistently had fewer NFR peaks than control samples in both cell types, indicating that BRG1 depletion resulted in a loss of accessible chromatin (Fig. 6E).

For downstream analysis, we trimmed the total number of peaks down to a high confidence set of 137,958 hESC NFR peaks and 164,265 NPC NFR peaks. These peak sets were merged into combined peak set of 186,627 NFR peaks for differential analysis. Differentially accessible NFRs were then defined as peaks with >2 fold change and an adjusted *P* < 0.01 between sets of biological triplicates. BRG1 depletion predominantly resulted in loss of accessibility at both hESC and NPC NFRs, with over 10-fold more “lost” NFRs than “gained” NFRs (Fig. 6, B and C). Comparatively, there were very few differential NFRs between BRG1KD and PROTAC conditions, indicating that the two distinct methods of BRG1 depletion had consistent effects on chromatin accessibility (Fig. 6, B and C). The effect of BRG1 depletion was more pronounced in NPCs, with >3-fold more differential NFRs in NPCs (Fig. 6, B and C). Thus, BRG1 was required to maintain accessible chromatin at NFRs in both hESCs and NPCs.

To assess the genomic context of the differential NFRs, we examined the distances between NFRs and annotated transcription start sites (TSSs). The genomic distribution of NFRs in hESCs and NPCs was very similar. With the total sets of NFRs at each time point, ~20% of peaks occurred within 1 kb of an annotated TSS, the remaining 80% of peaks had a broad distribution ranging from >1 kb to >1 Mb from the nearest TSS, and the median distance to the closest TSS was ~30 kb (Fig. 6F). In general, differential NFRs did not directly overlap TSSs (Fig. 6F). Despite this, the median distance to the closest TSS for NFRs that gained accessibility following BRG1 depletion remained ~30 kb (Fig. 6F). Conversely, NFRs that lost accessibility after BRG1 depletion trended much further away from TSSs, with a median distance of ~65 kb (Fig. 6F). For example, 37 NFRs were called in a 600-kb region surrounding the *CDH8* gene, which is down-regulated in BRG1KD NPCs (Figs. 3F and 6D). Accessibility was unaffected at the *CDH8* TSS, but the accessibility of many of the surrounding NFRs was markedly reduced following BRG1 depletion in both hESCs and NPCs (Fig. 6, D and G). The extent of accessibility changes at gained and lost NFRs also differed, with a greater magnitude of change in accessibility at lost NFRs (Fig. 6, H and I). Both gained and lost NFRs were marked by histone H3 lysine 27 acetylation (H3K27ac) enrichment in control cells, indicating that these NFRs likely represented active enhancers (Fig. 6, L and M). However, at the lost NFRs, BRG1 depletion resulted in near complete loss of H3K27ac enrichment, whereas enrichment was largely unchanged at gained NFRs (Fig. 6, L and M). Hence, BRG1 was required during the first 3 days of NPC differentiation to maintain accessibility and H3K27ac at thousands of active enhancers.

To gain insight into the potential regulation of these BRG1-dependent enhancers, we searched for enriched transcription factor motifs within the NFRs. The NFRs that lost accessibility after BRG1 depletion in NPCs were most strongly enriched for SOX motifs (Fig. 6J). The lost NFRs were also enriched for motifs of other transcription factors involved in neuroectodermal differentiation such as OTX2, ASCL1, and ZIC1 but were not enriched for PAX motifs (Fig. 6J). Conversely, NFRs that gained accessibility following BRG1 depletion were most strongly enriched for CTCF/CTCFL motifs and also enriched for KLF/SP1 motifs (Fig. 6K). Thus, BRG1 was critical for the maintenance of chromatin accessibility at enhancers likely involved in neuroectoderm differentiation.

### BRG1 establishes the NPC chromatin landscape

**Fig. 7.**
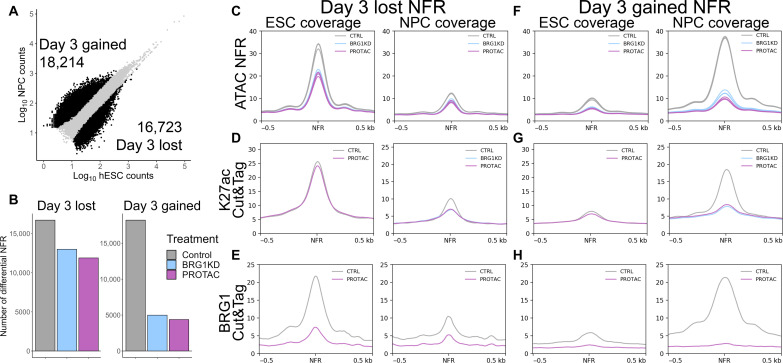
BRG1 establishes the NPC chromatin landscape. (**A**) Scatter plot comparing NFR counts between hESCs and NPCs over the total set of NFRs. Black dots and numbers represent NFRs with significantly more or less accessibility in NPCs versus hESCs. (**B**) Bar plots depicting the number of day 3 lost and day 3 gained NFRs in each hESC-NPC treatment pair. (**C**) Meta-profiles of hESC and NPC ATAC-seq NFR coverage over day 3 lost NFRs. (**D**) Meta-profiles of hESC and NPC H3K27ac CUT&Tag coverage over day 3 lost NFRs. (**E**) Meta-profiles of hESC and NPC BRG1 CUT&Tag coverage over day 3 lost NFRs. (**F**) Meta-profiles of hESC and NPC ATAC-seq NFR coverage over day 3 gained NFRs. (**G**) Meta-profiles of hESC and NPC H3K27ac CUT&Tag coverage over day 3 gained NFRs. (**H**) Meta-profiles of hESC and NPC BRG1 CUT&Tag coverage over day 3 gained NFRs.

Performing ATAC-seq at both day 0 and day 3 of NPC differentiation allowed us to examine chromatin accessibility dynamics between transitioning cell types. In control hESCs, 16,723 NFRs lost accessibility during early NPC differentiation, and 18,217 NFRs gained accessibility. We labeled these NFRs as day 3 lost and day 3 gained NFRs, respectively (Fig. 7A). The same statistical comparison in BRG1-depleted cells revealed a modest decrease in the number of day 3 lost NFRs and a greater decrease in the number of day 3 gained NFRs (Fig. 7B). This demonstrated that BRG1 was required for chromatin accessibility dynamics during NPC differentiation.

Despite lower levels of accessibility at day 3, BRG1 depletion reduced accessibility at day 3 lost NFRs in both hESCs and NPCs (Fig. 7C). H3K27ac was also decreased at day 3 lost NFRs in NPCs and even further decreased after BRG1 depletion (Fig. 7D). Thus, BRG1-dependent changes in accessibility and acetylation at day 3 lost NFRs were in the same direction as the changes that occurred during differentiation. We next reasoned that the loss of accessibility at these NFRs during differentiation was independent of BRG1. While BRG1 was enriched at these NFRs in hESCs, BRG1 enrichment was markedly reduced in NPCs (Fig. 7E). This indicated that BRG1 was not required for the loss of chromatin accessibility at NFRs that “closed” during NPC differentiation.

At day 3 gained NFRs, the requirement for BRG1 was more obvious. Again, accessibility was reduced at day 3 gained NFRs in both hESCs and NPCs (Fig. 7F). However, the differentiation-associated increase in accessibility at these NFRs was markedly reduced in BRG1-depleted cells (Fig. 7F). The day 3 gained NFRs had low levels of H3K27ac enrichment in hESCs and gained H3K27ac enrichment during differentiation (Fig. 7G). Conversely, BRG1 depletion blocked the accumulation of H3K27ac signal at day 3 gained NFRs (Fig. 7G). In accordance with the patterns of accessibility and acetylation, BRG1 was poorly enriched at these sites in hESCs and robustly enriched in NPCs (Fig. 7H). Thus, the differentiation-associated increases in chromatin accessibility and H3K27ac at day 3 gained NFRs were both dependent on and associated with BRG1 interaction. Together, these experiments demonstrated that BRG1 was required to “open” chromatin and activate neuroectodermal enhancers during NPC differentiation.

## DISCUSSION

The BAF complex is essential throughout many stages of mammalian development, and the integrity of the BAF complex is critical for human health (*4*, *6*, *43*). Specific requirements for the BAF complex in neural development have been demonstrated with a variety of genetic disruptions of individual BAF complex subunits (*31*). For example, neural differentiation was suppressed in hESCs lacking the BAF250A subunit, suggesting a restrictive role for the BAF complex (*30*). Additionally, we previously demonstrated that the core subunit BAF47 was required in hESCs for enhancer regulation and neuroectodermal differentiation (*23*). However, depletion of individual subunits does not disrupt the formation of the BAF complex, and, hence, the requirement for BAF complex function in neuroectodermal differentiation remained to be elucidated.

Here, we directly address the role of the BAF complex by depleting BRG1, the predominant catalytic subunit in hESCs. BRG1 depletion promoted up-regulation of genes enriched for activation of basic processes related to neural development including several transcription factors involved in neuroectodermal differentiation. During directed differentiation to NPCs, depletion of BRG1 disrupted the normal process of lineage specification. While control NPCs acquired a forebrain-like NPC fate, BRG1-depleted NPCs exhibited abnormal lineage trajectories. BRG1-depleted NPCs exhibited gene expression patterns associated with progenitor cells in the dorsal neural tube, were more prone to neuronal differentiation, and precociously gave rise to neural crest cells. These effects were only observed if BRG1 was depleted during the first 3 days of differentiation. Thus, BRG1 was required during NPC differentiation to prevent NPC dorsalization and inappropriate neural crest specification.

BRG1 depletion disrupted the normal expression patterns of NPC-enriched transcription factors such as *SOX2*, *PAX6*, *POU3F1*, *SOX21*, and *LHX5* and resulted in the formation of a heterogeneous NPC population with multiple distinct cell fates. BRG1-depleted NPCs predominantly adopted a dorsalized cell fate largely marked by expression of transcription factors such as *PAX3* and *ZIC1*. A neural crest cell population marked by expression of the transcription factors *SOX10*, *FOXD3*, *ETS1*, and *TFAP2A*/*B*/*C* also arose from BRG1-depleted NPCs. BRG1-depleted NPCs were also more prone to neuronal differentiation and formed a differential neuronal population with enhanced expression of the transcription factor *ASCL1*. Expression of these lineage-specific transcription factors likely drove the emergence of these cell populations. However, the NFRs lost after BRG1 depletion were also enriched for motifs of many of these factors. Furthermore, many of these transcription factors have been shown to recruit BRG1 and require BRG1 to function in a variety of contexts (*33*, *34*, *44*, *45*). Therefore, the role of these transcription factors in directing cell fate decisions following BRG1 depletion remains unclear. Directly interrogating their genomic distribution and requirement in the distinct NPC cell clusters could reveal how depletion of BRG1 alters their roles in regulating lineage-specific transcriptional programs.

In hESCs and day 3 NPCs, BRG1 was essential to maintain accessible chromatin at thousands of NFRs. The NFRs with BRG1-dependent accessibility tended to be distal from annotated TSSs and were marked by H3K27ac, indicating that they were likely active enhancers. Depletion of BRG1 reduced both accessibility and H3K27ac at BRG1-dependent sites, and these sites were strongly enriched for SOX motifs and motifs of other neuroectodermal transcription factors. Thus, it appeared that BRG1 was necessary to maintain accessibility and histone acetylation at enhancers involved in NPC differentiation. BRG1 depletion greatly reduced the number of genomic regions that gained accessibility during the first 3 days of NPC differentiation. More than 10,000 sites that gained accessibility and histone acetylation during differentiation were dependent on BRG1. Together, these findings demonstrated that BRG1 was required to open and activate chromatin at enhancers essential for normal NPC specification.

The enrichment of SOX family transcription factor motifs within BRG1-dependent NFRs suggested that BRG1 was vital for a SOX-driven transcriptional program during NPC differentiation. SOX transcription factors directly interact with BAF complexes in a variety of contexts including NPCs and other neural lineages. SOX factors can function as pioneer factors and bind to nucleosomal DNA to promote DNA accessibility for other factors (*46*). Other pioneer factors such as OCT4 and PU.1 require BRG1 to generate accessible chromatin (*47*, *48*). Hence, we propose a salient hypothesis for BRG1 function during NPC specification: Activation of NPC transcriptional programs is dependent on BRG1-mediated chromatin remodeling directed by SOX transcription factors.

The different effects of BRG1 and BAF47 depletion on NPC specification also provide insight into the regulation of BAF complex function during development. In contrast to the dorsalization we observe in BRG1-depleted NPCs, we and others have shown that BAF47 depletion generally impairs neural differentiation from hESCs (*23*, *49*). Despite varied reports on the role of BAF47 in the composition and integrity of the BAF complex, BAF47 is necessary for BAF complex interaction with enhancers, regulation of enhancer activity, eviction of Polycomb repressive complexes from chromatin, and activation of bivalent promoters (*50*–*55*). Thus, depletion of BAF47 impairs or alters BRG1 and BAF complex function. The underlying mechanisms of these differential effects remain unclear. BAF47-containing BAF complexes may have a distinct function in promoting the transition from pluripotency to differentiation. Alternatively, BAF complexes without BAF47 (either non-canonical ncBAF or BAF and PBRM1-containing PBAF complexes lacking BAF47) may have differential effects on chromatin accessibility and enhancer activity, and enrichment of these effects following BAF47 depletion could yield a distinct phenotype. These findings serve to demonstrate the intricacies and difficulties of targeting BAF complex activity. Hence, consideration must be given to context-dependent distinctions between different modalities for targeting BRG1 and the BAF complex for therapeutic purposes.

*SMARCA4* and other BAF subunit gene mutations have been identified in multiple aggressive pediatric brain tumors. ATRTs are commonly associated with mutations in *SMARCB1*, but a small and distinct subclass of ATRTs have biallelic mutations in *SMARCA4* (*18*, *20*). Tumors of this ATRT-*SMARCA4* subgroup have higher incidence of germline mutations, earlier age of incidence, and worse prognoses than the other subgroups with *SMARCB1* mutations (*18*, *20*). *SMARCA4* mutations are also frequently found in the WNT group and group III subgroups of medulloblastomas, with group III being the subgroup with the earliest age of incidence and worst prognoses (*56*, *57*). Both tumor types are thought to arise from neural progenitors in the midbrain, hindbrain, or neural crest. Only BRG1KD NPCs were classified with these cell type annotations when modeled on transcriptional profiles from first-trimester fetal human brains. Thus, we propose that biallelic loss of BRG1 expression or function in NPCs within the developing brain could promote the acquisition of inappropriate dorsal/caudal fates concomitant with tumor initiation or malignant conversion. This potential aberrant NPC specification could be a contributing factor to the aggressiveness and poor prognoses of *SMARCA4*-mutated brain tumors. BAF47 depletion at later stages of neural differentiation leads to failures in neuronal maturation and produces NPCs with a transcriptional profile similar to ATRTs (*49*). Tracking BRG1-depleted NPCs through later stages of differentiation to determine their ultimate cell fates could provide greater insight into the pathogenicity of *SMARCA4* mutations during embryogenesis.

## MATERIALS AND METHODS

### ESC cell culture and generation of BRG1KD hESC cell line

ESCs were cultured feeder-free in TeSR-E8 basal medium on Matrigel-coated tissue culture vessels and incubated in a 5% CO_2_ atmosphere and at 37°C. ReLeSR reagent was used to collect hESC aggregates during general passaging. Subcloning of the shRNA against *SMARCA4* was performed by digestion of the pINDUCER10 backbone vector with Xho I and Mlu I, following standard procedures as previously described (*23*, *58*). Lentiviruses carrying the respective shRNAs were produced at the National Institute of Environmental Health Sciences (NIEHS) Viral Vector Core Laboratory according to a previously established protocol (*59*). H1 cells were infected at a multiplicity of infection of 8 and selected using puromycin (1 mg/ml) for 24 hours. After an 18-hour Dox treatment (1 μg/ml), red fluorescent protein + HIGH cells were used to establish the shBRG1 cell line and used for all subsequent experiments. Dox treatment of hESC cultures for induction of shRNA and red fluorescent protein expression was performed using Dox (1 μg/ml) in TeSR-E8 medium for 3 days, unless otherwise stated. ACBI1 PROTAC treatment for targeted degradation of BRG1 was performed using 0.5 μM PROTAC in TeSR-E8 medium for 24 hours, unless otherwise stated.

### Monolayer neural induction protocol and human NPC culture

ESCs were cultured as described and collected as single-cell suspensions using the TrypLE Express reagent. Cells were then cultured according to the STEMCELL Technologies’ instructions for the STEMDiff Dual Neural Induction System for the generation of NPCs using dual SMAD inhibition. Briefly, hESCs were plated as single cells at a density of 2.0× 10^5^ cells/cm^2^ in STEMDiff Neural Induction Medium with SMADi, and 10 μM Y-27632 on Matrigel-coated plates and incubated in a 5% CO_2_ atmosphere at 37°C. Medium was changed daily with STEMDiff Neural Induction Medium with SMADi. In experiments where shRNA expression was induced using Dox or BRG1 protein degradation was induced using the PROTAC molecule, Dox or PROTAC was included in the medium throughout the experiment unless otherwise indicated. At specific experimental time points during differentiation, NPCs were collected using Accutase and further processed depending on the downstream application.

### Quantitative PCR

RNA was isolated from cells using RNeasy Plus kit purification with genomic DNA eliminator columns. cDNA was generated from total RNA using SuperScript III and either oligo(dT) or random hexamers. Quantitative PCR was run using ssoAdvanced Universal SYBR Green Supermix and gene/transcript specific primers. All experiments were performed with three or more biological replicates. Quantitative PCR data were normalized to control genes.

### Western blotting

Cells were lysed in TINE300 buffer [100 mM tris-HCl (pH 8.0), 1% IGEPAL CA-630, 300 mM NaCl, 1 mM EDTA, and 0.25 mM EGTA] containing 1:100 protease inhibitor cocktail and 1 mM phenylmethylsulfonyl fluoride. Cells were allowed to lyse for 20 min on ice, followed by 30 s of sonication. Lysate total protein concentrations were estimated by Bio-Rad protein assay. Total protein (20 to 40 μg) was applied per lane of acrylamide tris-glycine gels run at 125 V. Proteins were then transferred from gel to nitrocellulose membranes. Blots were then blocked for 30 min at room temperature in blocking solution (1× tris-buffered saline with 2% milk), followed by an overnight incubation in blocking solution plus primary antibodies. Blots were washed with wash buffer (1× tris-buffered saline with 1% Tween 20), followed by incubation in blocking buffer with species appropriate secondary antibodies. Blot imaging was performed using the LI-COR Odyssey imager.

### Flow cytometry

H1 hESC and NPC single cells were collected using dissociation protocols described above, washed with 1× phosphate-buffered saline and stained with fixable viability dye. Cells were washed with FACS buffer (1× phosphate-buffered saline, 2% bovine serum albumin, 1 mM EDTA, and 0.1% Na azide), followed by incubation in FACS buffer with fluor-conjugated surface antibodies for 2 hours at room temperature and with occasional mixing. Cells were washed with FACS buffer and incubated in fixation and permeabilization buffer at 4°C overnight. Fixed cells were washed with permeabilization wash buffer, followed by incubation in permeabilization wash buffer with intracellular target antibodies. Cells were washed with FACS buffer before analysis using a Becton Dickinson LSRFortessa flow cytometer. Flow cytometry data were processed using FlowJo v10. Debris, cell clusters, and permeability dye–positive cells were excluded from downstream analysis. Single-marker quantification was performed using histogram gating as compared to known positive populations and isotype controls (fig. S3A). Graphed data presented have mean values of 2 or more independent experiments with three or more biological replicates each.

### RNA sequencing

RNA-seq experiments were performed on three biological replicates per sample. RNA was isolated from hESCs using the total RNA purification kit, followed by ribosome RNA removal, library preparation, and sequencing by expression analysis, an IQVIA company. Transcript abundance was quantified using Salmon (*60*) with an index containing Gencode version v32lift37 comprehensive transcripts (*61*), human class I and II enhancers (*36*), and the hg19 human genome as decoy. Data were imported into R-3.6.1 for analysis and summarized to gene level with tximport (*62*), and then statistical contrasts were performed using limma-voom (*63*) moderated *t* tests.

### Assay for transposase-accessible chromatin with high-throughput sequencing

Single-cell suspensions of H1 hESCs or day 3 NPCs were generated as described above. Tagmentation was performed on 50,000 cells using Tn5 enzyme in tagmentation buffer on three biological replicates per condition. Isolation of libraries and amplification was performed as described (*64*). Paired-end reads were trimmed with cutadapt (*65*), aligned to hg19 with bowtie2 (*66*), and deduplicated with picardtools. Nucleosome free reads were defined with bamtools by keeping reads with insert sizes less than 101 bases. NFRs were identified by calling peaks with macs2 (*67*) with mfold limits of 10 and 200 and false discovery rate cutoff of 0.001. NFRs were merged with bedtools (*68*) and filtered to only keep NFRs that were called in more than one replicate of at least one treatment group. Nucleosome-free reads within NFRs were quantified with FeatureCounts (*69*), and differential analysis was performed with DESeq2 (*70*) with fold change > 2 and adjusted *P* < 0.01 cutoffs.

### Single-cell RNA sequencing

Single-cell suspensions were prepared for two biological replicates per cell type at concentration of 0.8 × 10^6^ to 1.2 × 10^6^ cells/ml and with cell viability of 80% or above. Briefly, approximately 5000 cells were used together with the Chromium Single Cell 3′ Library & Gel Bead Kit v3.1 onto the 10x single-cell chromium chip to generate a single-cell emulsion in Chromium Controller. Reverse transcription of mRNA and cDNA amplification was carried out following the manufacturer’s instruction. The amplified cDNA was further fragmented to construct next-generation sequencing libraries. The libraries were then sequenced by the NIEHS Epigenomics and DNA Sequencing Core Laboratory with the parameters recommended in the manufacturer’s instruction. Fastq files were processed with CellRanger v3.0.1 using GRCm38 genome and Gencode annotation provided by 10x Genomics to generate the initial cell by gene matrix for each sample. Matrices were read into Seurat v4 (*71*) using R-4.2.1 and merged into a single Seurat object. Cells with greater than 10% mitochondrial reads or less than 4000 UMI were removed from the object. UMAP dimensional reduction was performed using the top 15 principal components, and clusters were called with resolution = 1.5. From this object, the raw counts matrix was exported for cell annotation using the online tool at celltypist.org. Data were visualized with built-in Seurat tools or with ggplot2.

### CUT&Tag

CUT&Tag was largely performed as per Epicypher and Henikoff laboratory protocols. Briefly, nuclei were isolated, fixed for 2 min with 0.1% formaldehyde at room temperature, and cryopreserved before all CUT&Tag experiments. For each experiment, nuclei were thawed, and 100,000 nuclei were used per reaction. Nuclei were bound to activate concanavalin A beads, and 0.5 μg of primary antibody was bound overnight at 4°C. Secondary antibody was bound for 30 min at room temperature, pAG-Tn5 was bound for 1 hour at room temperature, and the tagmentation reaction was incubated at 37°C for 1 hour. Libraries were amplified with NEBNext high-fidelity PCR mix, cleaned up with AMPure XP beads, and sequenced by the NIEHS Epigenomics and DNA Sequencing Core Laboratory. Paired-end reads were trimmed with cutadapt (*65*), aligned to hg19 with bowtie2 (*66*), and deduplicated with picardtools. Coverage files and meta-profiles were generated with DeepTools (*72*).

### Statistical analysis

Statistical analyses were performed using R or GraphPad Prism software. Sample sizes (*n*) and *P* value calculations are described in legends for each figure.
