# Supplementary material for: BRG1 establishes the neuroectodermal chromatin landscape to restrict dorsal cell fates
Source: Sci Adv. 2024 Mar 1;10(9):eadj5107. doi: 10.1126/sciadv.adj5107 (PMC10906928; doi:10.1126/sciadv.adj5107)
Supplement: Supplementary file 1 — Figs. S1 to S6 Tables S1 and S2 [file sciadv.adj5107_sm.pdf]

Science Advances
AAAS

Supplementary Materials for

BRG1 establishes the neuroectodermal chromatin landscape to restrict dorsal cell fates

Jackson A. Hoffman *et al.*

Corresponding author: Trevor K. Archer, archer1@niehs.nih.gov; Jackson A. Hoffman, jackson.hoffman@nih.gov

Sci. Adv. **10**, eadj5107 (2024)
DOI: 10.1126/sciadv.adj5107

This PDF file includes:

Figs. S1 to S6
Tables S1 and S2

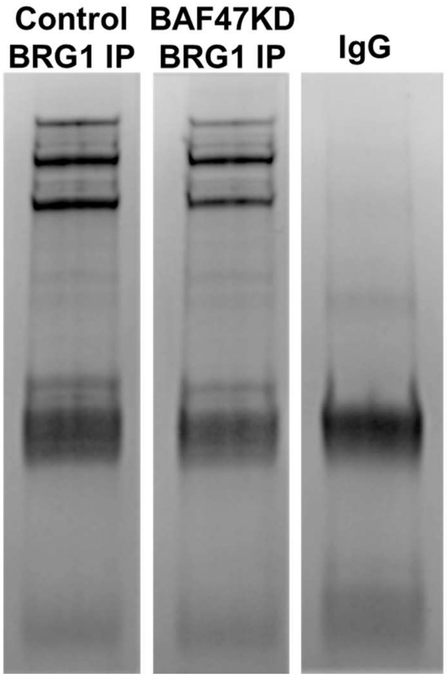

Ctrl	BAF47KD	symbol
440	440	BRG1
397	295	BAF155
285	135	BAF60A
145	93	ACTG1
143	78	BAF53A
42	22	BCL7C
605	388	BAF250A
69	35	BAF45D
4	0	BAF45B
162	100	BAF57
96	31	BAF47
122	128	BAF180
91	141	BAF200
42	16	BRD7
15	1	BAF45A
56	79	GLTSCR1
33	27	BRD9
9	1	GLTSCR1L

Figure S1: BAF47-independent formation of BAF complexes. Coomassie stained images of BRG1 or IgG immunoprecipitations from control or BAF47KD ESC. Table shows relative number of spectra detected for BAF complex subunits. Spectra counts normalized to number of BRG1 spectra.

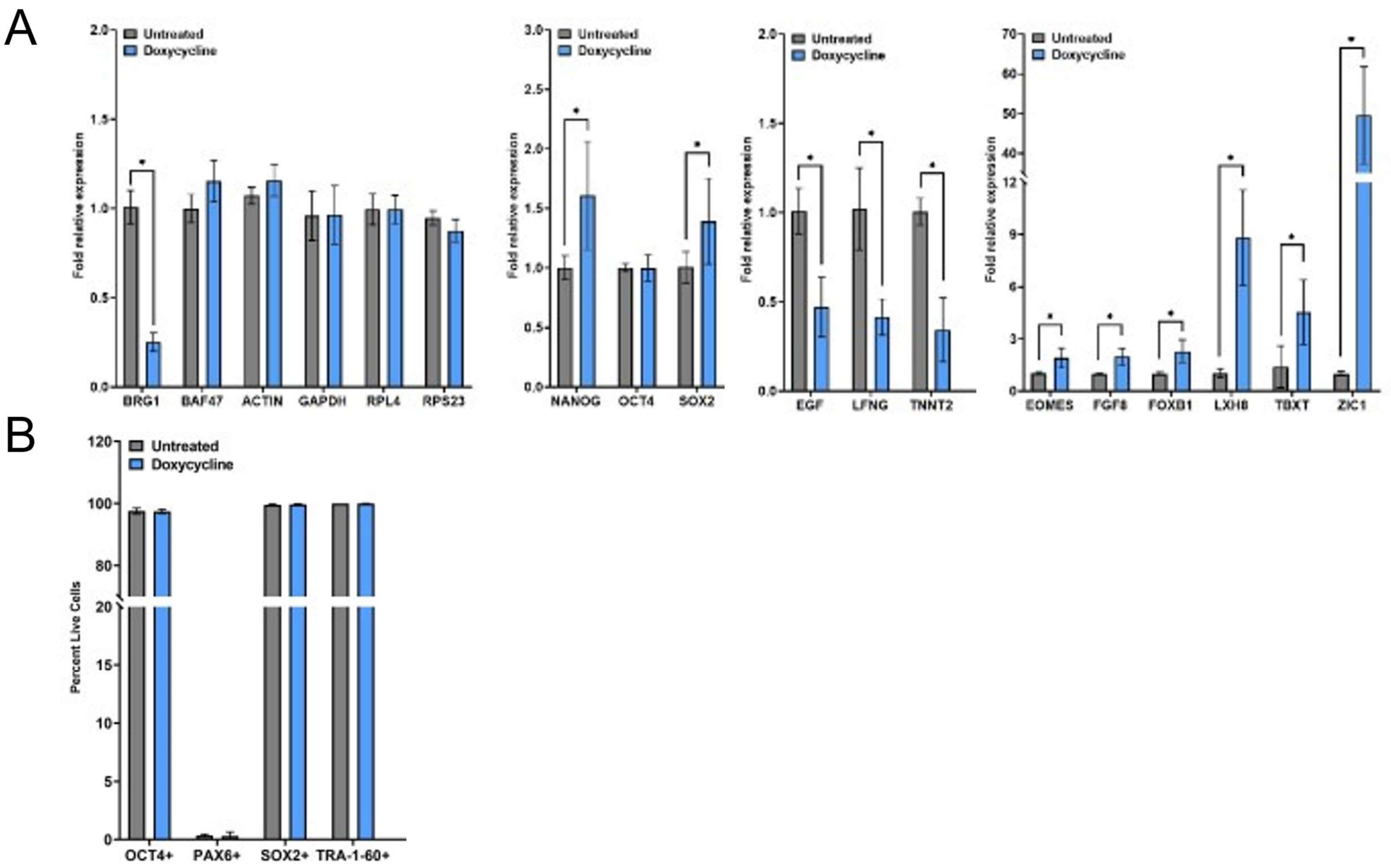

Figure S2: Altered gene expression in BRG1KD ESC. A) RT-PCR for BAF subunits, pluripotency genes, down-regulated RNA-seq DEGs, and up-regulated RNA-seq DEGs. B) Percent of control or BRG1KD ESC positive for the listed markers.

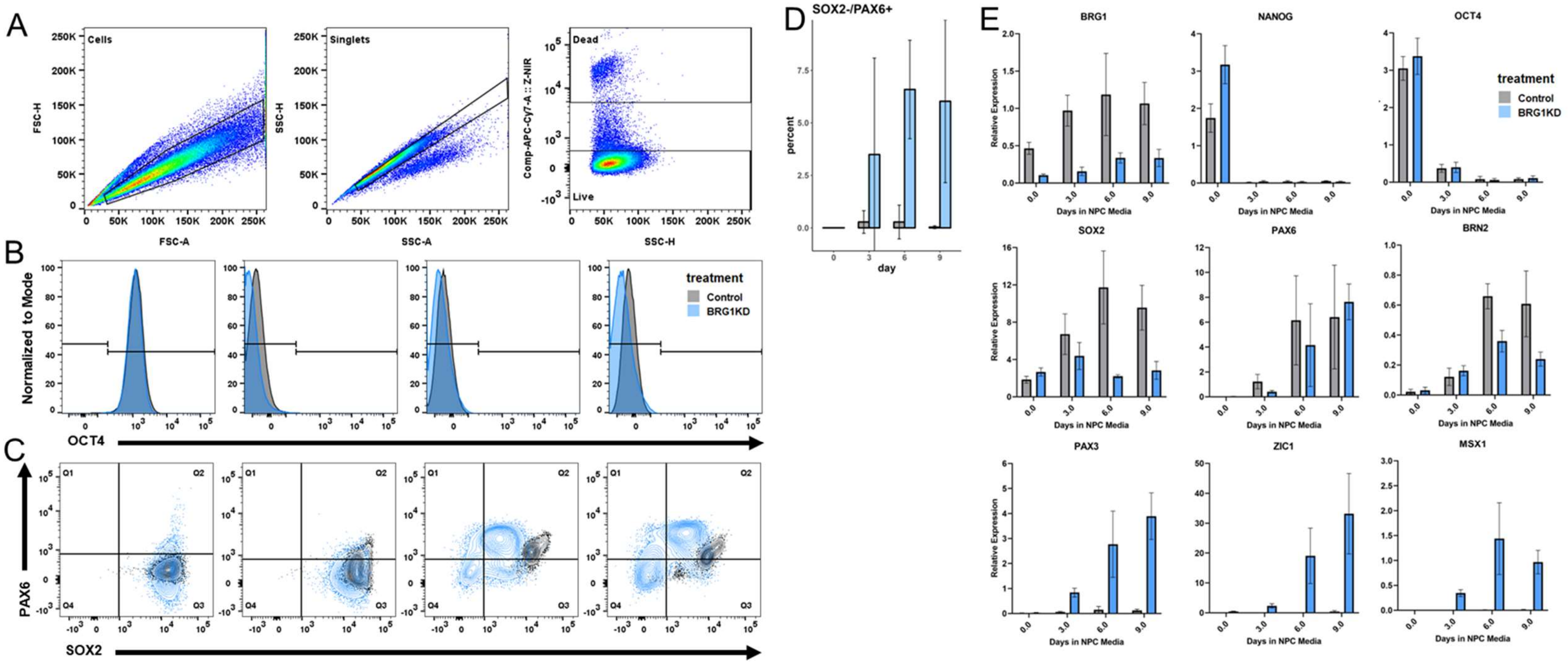

Figure S3: Altered protein and gene expression in BRG1KD NPCs. A) FACS gating parameters. B) FACS histogram of OCT4 protein detection at collection timepoints. Log10 fluorescent signal depicted for control cells in gray and BRG1KD cells in light blue. Data represents the total of all biological replicates (n >= 3). C) FACS contour plot of SOX2 and PAX6 protein detection at collection timepoints. Contour lines represent 2% intervals of cell density, control cells in gray and BRG1KD cells in light blue. Data represents the total of all biological replicates (n >= 3). D) Graph depicting the percent of cells at each collection timepoint that were SOX2-/PAX6+. Bar height indicates mean percentages and error bars represent standard deviation of biological replicates (n >= 3). Control values in gray, BRG1KD values in light blue. E) RT-PCR for BRG1, pluripotency markers, and neural transcription factors. Bars depict mean expression level relative to control genes; error bars represent standard deviation of biological triplicates.

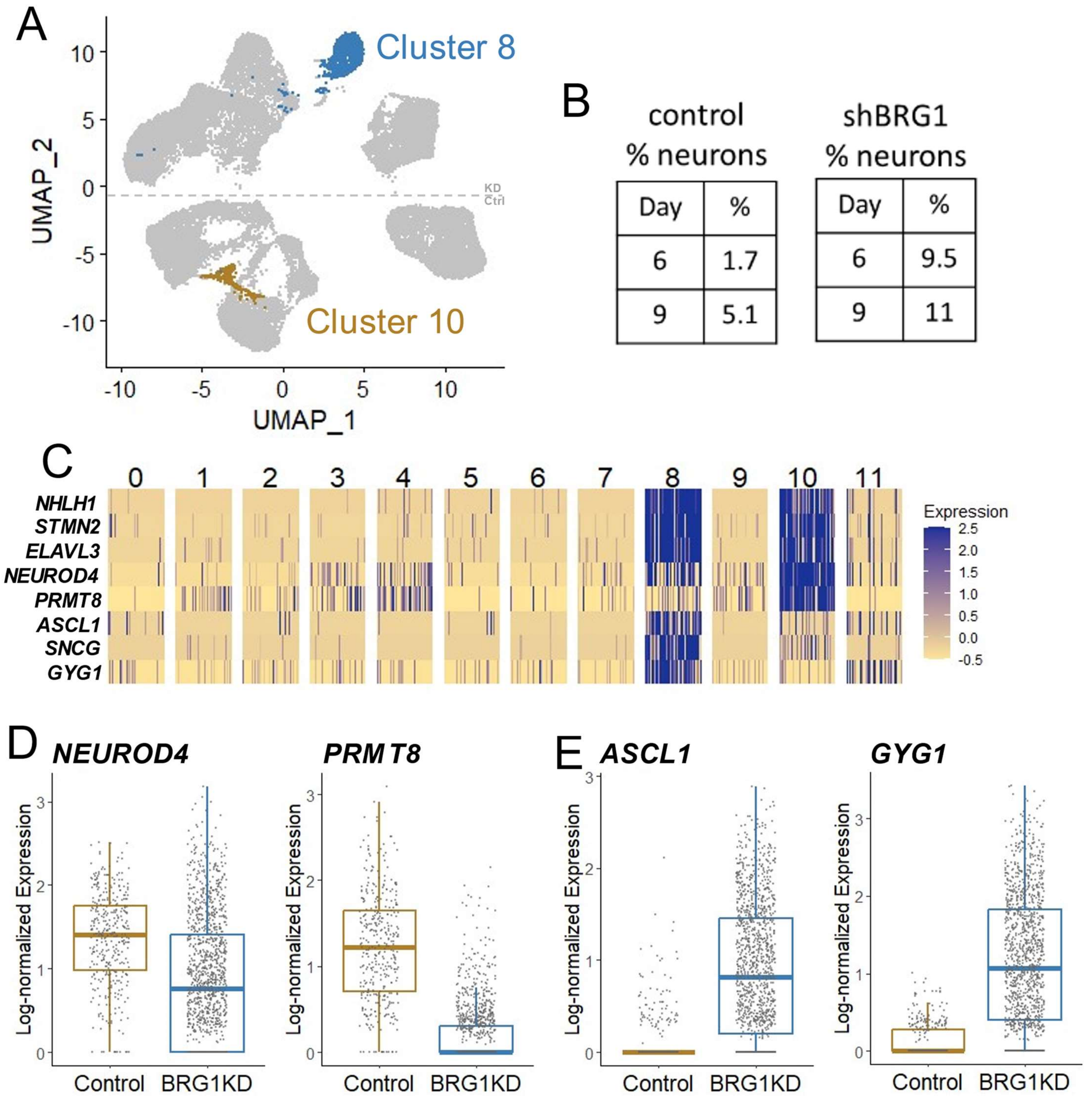

Figure S4: Altered neuronal specification in BRG1-depleted NPCs. A) UMAP plot highlighting clusters 8 and 10. B) Percentages of control and BRG1KD cells in clusters 10 and 8, respectively. C) Heatmap of log-scaled expression of neuronal marker genes in randomly down-sampled Seurat clusters (50 cells per cluster). D,E) Box and jitter plots of neuronal marker genes differentially expressed between clusters 8 and 10.

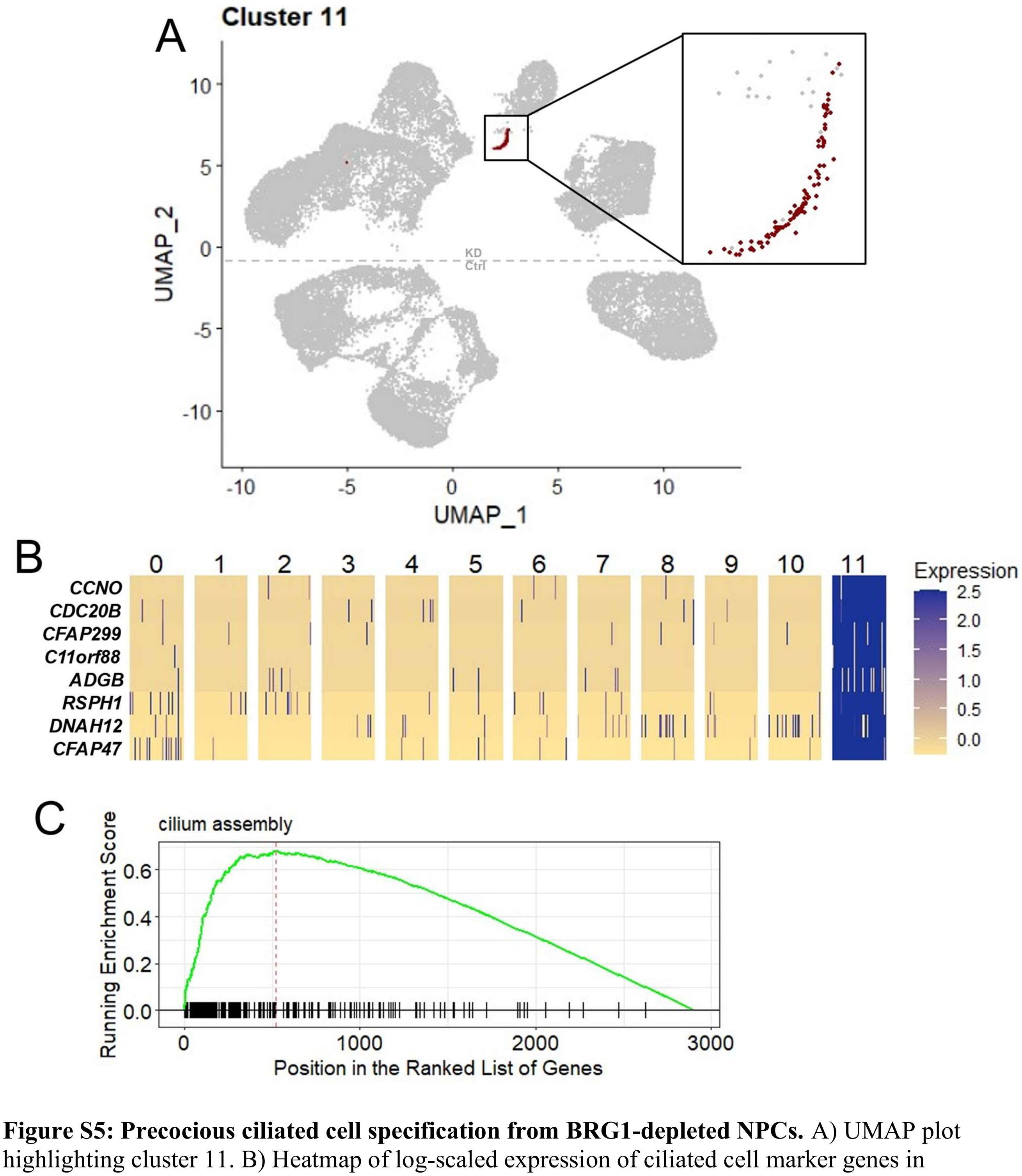

Figure S5: Precocious ciliated cell specification from BRG1-depleted NPCs. A) UMAP plot highlighting cluster 11. B) Heatmap of log-scaled expression of ciliated cell marker genes in randomly down-sampled Seurat clusters (50 cells per cluster). C) GSEA plot of running enrichment score for “cilium assembly” gene set within assigned marker genes of cluster 11.

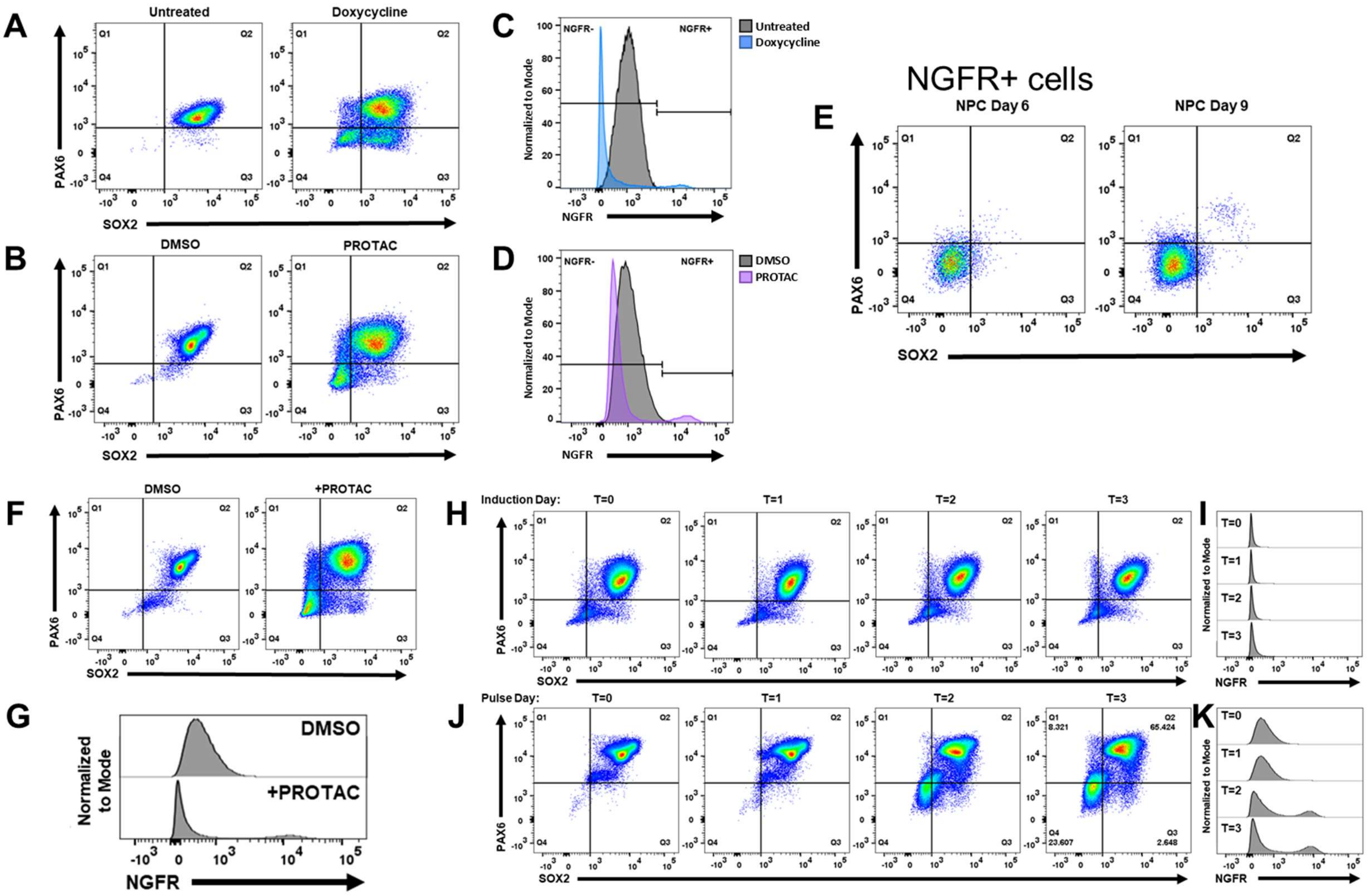

Figure S6: FACS scatter plots corresponding to Figure 5. A,B,F,H, and J) Cell scatter plots depicting levels of SOX2 and PAX6 protein expression. Color indicates density of points; blue = low, red = high. C,D) FACS histograms depicting the density of cells expressing NGFR protein. E) Cell scatter plots depicting levels of SOX2 and PAX6 protein expression in NGFR+ BRG1KD NPCs at days 6 and 9. Color indicates density of points; blue = low, red = high. G, I, and K) FACS histograms of NGFR protein expression.

Table S1: Oligonucleotide Sequences

Gene	Forward Primer	Reverse Primer
ACTB	ACCTTCTACAATGAGCTGCG	CCTGGATAGCAACGTACATGG
TUBA1B	TTGTAGACTTGGAACCCACAG	ATCTCCTTGCCAATGGTGTAG
GAPDH p1	ACATCGCTCAGACACCATG	TGTAGTTGAGGTCAATGAAGGG
ACTIN	GGCACCCAGCACAATGAAGATCAA	ACTCGTCATACTCCTGCTTGCTGA
BAF47	GGATGGCAACGATGAGAAGT	TGTTGATGGTTGTGGAGCAT
EGF	TGTCCACGCAATGTGTCTGAA	CATTATCGGGTGAGGAACAACC
EOMES	CTGGCTTCCGTGCCCACGTC	CATGCGCCTGCCCTGTTTCG
FGF8	GGTCCTGGCCAACAAGCGCA	CCGTCTCGGCTCCTCGGACT
FOXB1	CTCTCCTTCAACGACTGCTTCA	GTGGTCGGACTTAAGCACCTT
GAPDH p2	TCTCCTCTGACTTCAACAGCGACA	CCCTGTTGCTGTAGCCAAATTCGT
LFNG	GCTGTCAAGACCACCAAAAAGTT	CCTCATCTTCCCCGTCAGTG
LXH8	GCTGTCTCCACCCATGTTAGAA	ATTGAATGGGGTAACAAGGGCT
RPL23	ACATCCAGCAGTGGTCATTCGACA	TCTGCACACTCCTTTGCTACTGGT
RPL4	TTCGAGCACCACGCAAGAAG	CCTTATCCACCCGGAGCTTG
TBXT	TATGAGCCTCGAATCCACATAGT	CCTCGTTCTGATAAGCAGTCAC
TNNT2	GCCCAATGGAGGAGTCCAAAC	CTGTTCTCAAAGTGAGCCTCG
BRG1	GCCGTGATCAAGTACAAGGAC	TATCTTCTTGAAGTCCACGGGC
NANOG	AATACCTCAGCCTCCAGCAGATG	TGCGTCACACCATTGCTATTCTTC
OCT4	GAGAACCGAGTGAGAGGCAACC	CATAGTCGCTGCTTGATCGCTTG
SOX2	TACAGCATGTCCTACTCGCAG	GAGGAAGAGGTAACCACAGGG
ZIC1	GCGCGCTCCGAGAATTTAAAGAT	ACGTGCATGTGCTTCTTGCG
BRN2	GCGGATCAAACTGGGATTTAC	GCACATGTTCTTGAAGCTCAG
MSX1	GAAGATGCGCTCGTCAAAGC	CTTACGGTTCGTCTTGTGTTTG
PAX3	ACAACGCCTGACGTGGAG	ATTTACTTCTCAGGATGCGGCT
PAX6	GCCCCATATTCGAGCCCCGT	TGGCCGCCCGTTGACAAAGA
FEZF1	CTGTGGCAAAGGGTTTCATC	GTGTGCATGTGGAAGGTGAG

Table S2

REAGENT or RESOURCE	SOURCE	IDENTIFIER
**Antibodies**		
BRG1	Santa Cruz Biotechnologies	sc-17796, RRID:AB_626762
BRG1	Novus Biologicals	NB100-2594, RRID:AB_2191852
Actin	Santa Cruz Biotechnologies	sc-10731, RRID:AB_2223515
SOX2	Millipore	AB5603, RRID:AB_2286686
PAX3	DSHB	Pax3, RRID:AB_2315061
LHX5	DSHB	PCRP-LHX5-1B7, RRID:AB_2722232
BAF60A	BD Biosciences	611728, RRID:AB_2192143
Beta-tubulin	DSHB	E7, RRID:AB_528499
OCT4-conjugated	BD Biosciences	560329, RRID:AB_1645318
SOX2-conjugated	BD Biosciences	562139, RRID:AB_10897844; 561593, RRID:AB_10894382
PAX6-conjugated	BD Biosciences	562388, RRID:AB_11153319
NGFR-conjugated	BD Biosciences	562562, RRID:AB_2737657
H3K27ac	Abcam	ab4729, RRID:AB_2118291
Anti-Rabbit CUT&Tag Secondary	Epicypher	13-0047
IRDye 800CW Donkey anti-Rabbit IgG	LI-COR Biosciences	926-32213, RRID:AB_621848
IRDye 680RD Donkey anti-Rabbit IgG	LI-COR Biosciences	926-68073, RRID:AB_10954442
IRDye 800CW Donkey anti-Mouse IgG	LI-COR Biosciences	926-32212, RRID:AB_621847
IRDye 680RD Donkey anti-Mouse IgG	LI-COR Biosciences	926-68072, RRID:AB_10953628
**Chemicals, peptides, and recombinant proteins**		
Formaldehyde Solution, 16%	ThermoFisher Scientific	28906
TeSR-E8 Basal Medium	STEMCELL Technologies	05990
hESC Matrigel	Corning	354277
ReLeSR reagent	STEMCELL Technologies	05872
Y-27632	STEMCELL Technologies	72304
TrypLE Express reagent	Gibco	12604013
STEMdiff™ SMADi Neural Induction Kit	STEMCELL Technologies	08581
ACBI1 PROTAC	MedChemExpress	HY-128359
BRM/BRG1 ATP Inhibitor-1	MedChemExpress	HY-119374
Doxycycline	Alfa Aesar	J60579
HALT Protease Inhibitors	ThermoFisher Scientific	78430
TWEEN 20	Millipore Sigma	P7949
CUTANA pAG-Tn5	Epicypher	15-1017
NEBNext High-Fidelity 2X PCR Master Mix	New England BioLabs	M0541L
**Critical commercial assays**		
Zombie NIR Fixable Viability Kit	BioLegend	423105
eBioscience Foxp3 / Transcription Factor Staining Buffer Set	ThermoFisher Scientific	00-5523-00
AMPure XP beads	Beckman Coulter	A63881
SuperScript III First-strand kit	ThermoFisher Scientific	18080-051
ssoAdvanced Universal SYBR Green Supermix	Bio-Rad	172-5274

RNeasy Mini Kit	Qiagen	74104
QiaQuick PCR Purification Kit	Qiagen	28104
Dounce Homogenizer	Duran Wheaton Kimble	357542
Protein A/G Dynabeads	ThermoFisher Scientific	88802
Bio-Rad Protein Assay Kit II	BIO-RAD	5000002
Total RNA Purification Plus Kit	Norgen Biotech	48300
Tagment DNA TDE1 Enzyme Kit	Illumina	20034197
Chromium Single Cell 3' Library & Gel Bead Kit v3	10X Genomics	1000268
BioMag Plus Concavalin A Beads	Bangs Laboratories Inc.	BP531
**Deposited data**		
GSE235534	This Study	GSE235534
**Experimental models: cell lines**		
H1 human embryonic stem cells	WiCell	WA01
**Oligonucleotides**		
See table S1		
**Software and algorithms**		
Cutadapt 1.9.1	Martin et al. (65)	RRID:SCR_011841
Bowtie2 2.0.0-beta7	Langmead and Salzberg (66)	RRID:SCR_016368
Samtools 0.1.20	https://www.htslib.org/	RRID:SCR_002105
Picard-tools 1.110(1752)	https://broadinstitute.github.io/picard/	RRID:SCR_006525
MACS2 2.1.1.20160309	Zhang et al. (67)	RRID:SCR_013291
Bedtools 2.24.0	Quinlan and Hall (68)	RRID:SCR_006646
Deeptools 2.5.3	Ramirez et al. (72)	RRID:SCR_016366
ggplot2 3.4.0	https://ggplot2.tidyverse.org	RRID:SCR_021139
FlowJo	BD Biosciences	RRID:SCR_008520
Salmon 1.3.0	Patro et al. (60)	RRID:SCR_017036
Tximport 1.14.2	Soneson et al. (62)	RRID:SCR_016752
Limma-voom 3.42.2	Ritchie et al. (63)	RRID:SCR_010943
featureCounts 1.5.0-p2	Liao et al. (69)	RRID:SCR_012919
DESeq2 1.38.3	Love et al. (70)	RRID:SCR_015687
CellRanger 3.0.1	10X Genomics	
Seurat 4.3.0	Hao et al. (71)	RRID:SCR_016341
R 3.6.1, 4.2.1, 4.2.2, 4.2.3, and 4.3.1		
